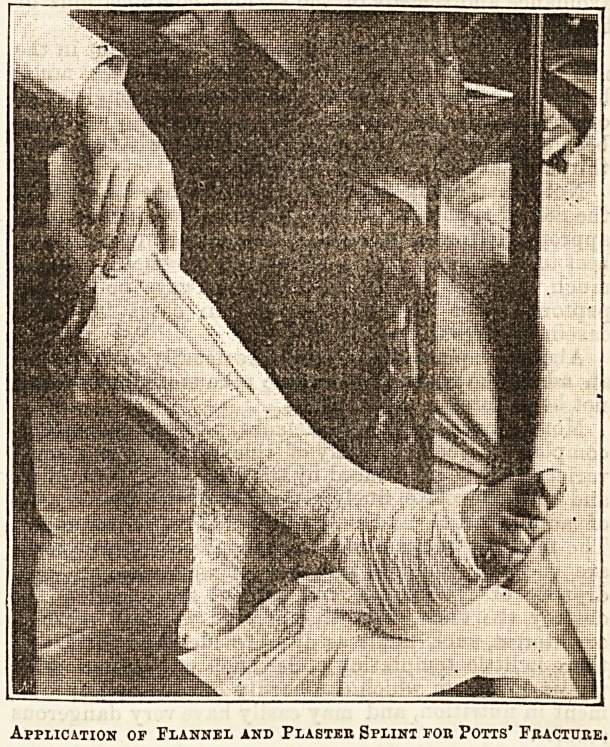# Treatment of Fractures of the Leg

**Published:** 1892-11-05

**Authors:** 


### HULL ROYAL INFIRMARY.

### Treatment of Fractures of the Leg.

The usual method of treating fractures of the leg at the Hull Royal Infirmary is by putting the limb up in back and side splints for three weeks, and then in a form of plaster of Paris splint till the tenth or twelfth week. In the case of a fracture of the upper half of the tibia, the back splint extends one-third of the distance up the thigh. The usual splint is a well-padded one, composed of thin sheet-iron slightly bent into a hollow from side to side, and also bent at the knee, to allow of comfortable flexion of the limb. An oval opening is cut in the iron at the site of the heel, and the end of the splint is turned up at right angles to form the foot-piece. By using thin iron as a material for the splint, an appropriate bend can be given to it to suit a case of fracture with anchylosed knee or ankle.

If the site of the fracture is below the middle of the tibia, the back splint need not extend higher than the bend of the knee.

The limb is placed upon the splint, and the fracture having been reduced, the limb is kept in position by strapping, and a bandage applied above and below, leaving the site of the fracture exposed. Wooden side splints with foot-pieces are then applied, and kept in position by webbing straps above and below. The leg is then slung in a cradle, and an ice-bag rests on a piece of lint over the site of the fracture. In the case of a compound fracture the wound is syringed well with perchloride of mercury lotion, 1-1,000, iodoform dusted into it, and well rubbed into the surrounding skin. Sublimate gauze and wool dressing is applied, a bracketed side splint being used if the bulk of the dressing requires it.

At the end of ten days, when swelling has disappeared, and blisters, if present, have healed, the limb is put up in an adjustable casing of flannel and plaster of Paris. These splints take about a quarter of an hour to apply—very little longer than it takes to apply the muslin plaster bandages, and no bandages rubbed with plaster are required.

The flannel and plaster splints are made in accordance with the method advocated by Mr. Croft, of St. Thomas's Hospital. The casing consists of two lateral splints, each made of an inner layer of plain house-flannel, and an outer layer of flannel which has been soaked in cream of plaster of Paris.

The fresh flannel when put into water shrinks to an appreciable extent, it is therefore kept ready shrunk for use.

To apply the splint, four pieces of flannel are first cut out, a guide to the size being furnished by a stocking the size of the patient's leg, care being taken to cut the foot-piece at right angles to the leg. If the fracture is situated above the middle of the tibia the splint should extend one third of the distance up the thigh; if the fracture is below the middle third the splint should extend as high as the head of the fibula, so as to allow of comfortable flexion of the knee. The

Application of Flannel and Plaster Splint for Potts' Fracture.

cut flannels are now applied to the sound leg to see that the fit is satisfactory, the edges at the back and front should be separated by an interval of half-an-inch. The skin of the fractured leg is now rubbed with a little oil or vaseline to prevent irritation from the coarse flannel, and a mackintosh or sheet of newspaper laid on the bed under the limb. A medium-sized basin is then half-filled with fresh plaster and warm water poured in gently until the flattened surface of the plaster is well-covered. In about a minute, when the bubbles have ceased to rise, the plaster is mixed into a cream and two of the cut flannels are rapidly drawn into the cream in succession, allowed to take up as much as possible, and are then laid on the two other flannels, which have been placed flat on the table with the toes facing each other. These are now applied to the limb with the flannel side inwards, and a broad bandage of stout muslin wrung out of hot water is put on without tension, from the toes, over the point of the heel and up to the top without reversing, the limb being meanwhile well supported so as not to disturb the fracture. The foot is kept at right angles until the plaster is well set, an unpadded back splint being bandaged on for a few hours if necessary to guard against movement. When the plaster is dry the wooden splint is removed. The surface of the muslin can be improved by rubbing it with a little starch mucilage or chalk mixed to a cream with gum mucilage. To prevent this getting on to the line along which the splint is to be opened up, a strip of strapping half-an-inch broad is carried along the line of junction of the flannels down the front and along the centre of the sole. This is removed after the application of the mucilage, and the heel is supported on an upturned basin until the splint is dry.

When it is desired to inspect the leg the splint is opened by running a blunt pointed pair of scissors along the front and down the centre of the sole to divide the bandage. The splint need not be removed, but by turning the leg on one side, one-half of the splint supports the fracture, while the other half is reflected on the back as a hinge.

In the case of a compound fracture a small dressing is placed on the wound before the splint is applied. This is renewed when the splint is opened by turning back the exposed half of the dressing, and applying half of the new dressing. The open half of the splint is then closed up, the leg turned on the other side, and supported on the closed half, while the old dressing is removed, and the free half of the new dressing applied, and the splint closed.

If frequent dressings are necessary the edges of the splint are covered with a "binding" of strapping, and an ordinary bandage applied. If the splint is to be kept closed a muslin bandage is applied, and a fresh layer of starch mucilage.

In adults the thin muslin is liable to give way over the front of the knee, so crinoline muslin is used as being stronger for the long splints. This splint lasts the patient till the end of the twelve weeks' rest that is usually necessary to secure firm union of the fracture.

This form of splint is also applied in cases of recent fracture, in delirious or violent patients, after osteotomy, or simple fracture of curved tibiæ in young children, in adults where anchylosis of knee or ankle prevents the application of the ordinary back splint, in oblique fracture of the tibia where the fragments cannot be otherwise prevented from "riding," and also in the case of curved tibia in an adult when there is an appreciable gap made between the fragments when the ball of the great toe, inner malleolus and inner border of the patella are placed in a straight line. By the flannel splint the fragments can be put up in an accurate position and no gap is left to delay union, and no alteration is made in the position of the bearing points of the foot. The condition of the limb is carefully watched after the application of the splint for recent injury. By its uniform pressure subcutaneous hæmorrhage is checked, and repair begins earlier than when much effusion has to be absorbed.

Should there be much pain, swelling, lividity, or coldness of the exposed part of the foot, the splint is at once cut open, and readjusted, or a new one applied. The limb is always inspected on the tenth day, so that the position of the fragments can be rectified if necessary before actual repair has commenced.

## Figures and Tables

**Figure f1:**